# Long noncoding RNAs that respond to *Fusarium oxysporum* infection in ‘Cavendish’ banana (*Musa acuminata*)

**DOI:** 10.1038/s41598-017-17179-3

**Published:** 2017-12-05

**Authors:** Wenbin Li, Chunqiang Li, Shuxia Li, Ming Peng

**Affiliations:** Key Laboratory of Biology and Genetic Resources of Tropical Crops, Institute of Tropical Bioscience and Biotechnology, Chinese Academy of Tropical Agricultural Sciences, Haikou, Hainan China

## Abstract

Long noncoding RNAs (lncRNAs) are a class of genes that influence a variety of biological functions through acting as signal, decoy, guide, and scaffold molecules. In banana (*Musa* spp.), an important economic fruit crop, particularly in Southeast Asia, the wilt disease caused by *Fusarium oxysporum* f. sp. *cubense* (*Foc*), especially strain *Foc* TR4, is disastrous. In banana, how the biogenesis of these lncRNAs is regulated in response to pathogen infection is still largely unknown. In this study, strand-specific paired-end RNA sequencing of banana samples was performed on susceptible and resistant cultivars inoculated with *Foc*, with three biological replicates and at two different times after infection. Overall, 5,294 lncRNAs were predicted with high confidence through strict filtration, including long intergenic ncRNA (lincRNA) and antisense lncRNA. Differentially expressed (DE) lncRNAs were identified in response to *Foc* infection in the inoculated versus the mock-inoculated banana of the susceptible ‘BX’ and resistant ‘NK’ cultivars. Through KEGG, GO, and the expression levels of the DE lncRNAs, some DE lncRNAs were predicted to be involved in plant-pathogen interactions and phytohormone signal transduction. In this study, this catalog of lncRNAs and their properties will facilitate further experimental studies and functional classifications of these genes.

## Introduction

Non-protein-coding RNAs (ncRNAs) comprise a substantial portion of the transcribed sequences within a genome and play important roles in a wide range of biological processes. Over the past few years, microRNAs (miRNAs), small interfering RNAs (siRNAs), and natural antisense siRNAs (nat-siRNAs) have been found to be involved in the transcriptional and post-transcriptional regulation of genes^1,2^. Non protein-coding RNAs longer than 200 nucleotides, or long non-coding RNAs (lncRNAs) are associated with virtually every biological process in plants, including plant development and response to biotic or abiotic stresses^3–7^. LncRNAs are the most common ncRNAs, but at this point are also the least understood lncRNAs in mammals and plants.

Based on its location and orientation to the nearest protein-coding transcripts, a lncRNA is classified as either intergenic, antisense, sense overlapping, sense intronic, or processed transcripts^8,9^. LncRNAs influence physiological and biochemical processed of plants by acting as molecular signals, decoys, guides or scaffolds^6^. In particular, long intergenic ncRNAs (lincRNAs) are key regulators of diverse cellular processes. Because of these important biological roles, lncRNAs have been of great research interest in recent years. Recent evidence from whole genome, RNA sequencing (RNA-seq), and computational methods have allowed for systematic identification and classification of lncRNAs in many plant species. For instance, 125 putative stress-responsive lncRNAs have been identified in wheat^10^; 504 lincRNAs were drought responsive in *Populus trichocarpa* ^11^; 931 lncRNAs were identified in response to *Sclerotinia sclerotiorum* infection in *Brassica napus* ^12^. Currently, the identification of lncRNA sequences far outpaces the understanding of their functions, although investigations into the cellular functions of individual lncRNAs in plants have been undertaken. For instance, several lncRNAs (i.e. *COLDAIR*, *COOLAIR*, *LDMAR*, *CsM10* and *Zm401*) have been demonstrated to participate in reproductive regulation^13–17^; five novel intergenic lncRNAs responsive to *Fusarium oxysporum* were characterized in disease development in *A*. *thaliana* ^18^; and *slylnc0049* and *slylnc0761* were characterized to play functions in the tomato yellow leaf curl disease^19^.

*Fusarium oxysporum* is a soil-borne fungal plant pathogen that causes either wilt of root, bulb or foot rot in a wide variety of plant species. Host-specific forms afflict many economically important crops such as tomato^20^, watermelon^21^ and potato^22^. *F. oxysporum* f. sp. *cubense* (*Foc*) causes wilt disease in banana (*Musa*. spp), particularly Tropical Race 4 (TR4), which causes drastic economic losses in the commercially grown cultivar ‘Cavendish’ throughout the banana producing areas of the world^23^. It is unknown whether lncRNAs participate in the *Foc* defense network in banana. No pathogen-responsive lncRNAs have been documented in banana so far. The resistant cultivar ‘Nongke No 1’ i.e. (‘NK’), a mutant of ‘Cavendish’ banana^24,25^, provides a chance to gain insights into the response of lncRNAs during *F. oxysporum* infection of banana.

In this study, we performed whole transcriptome strand-specific RNA sequencing to investigate the changes of lncRNAs during *Foc* TR4 infection in the roots of resistant ‘NK’ and susceptible ‘Brazil’ (‘BX’) banana cultivars. Furthermore, lncRNAs involved in plant-pathogen interactions, biosynthesis and transduction of auxin, ethylene, salicylic acid (SA) and jasmonic acid (JA), and the regulation of pathogenesis-related (PR) genes were studied in both cultivars. The banana genome database (http://banana-genome.cirad.fr/)^26^ allowed systematic identification of banana lncRNA (including lincRNAs and antisense lncRNAs). Transcriptome analysis was used to reveal the expression profiles of lncRNAs in different banana cultivars in response to *Foc* infection and to identify lncRNAs related to antifungal resistance in banana.

## Results

### Symptoms of infected plants

**Figure 1:**
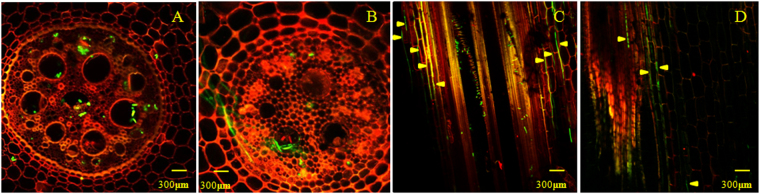
Examination of the infection process using GFP-expressing *Fusarium oxysporum* f. sp. *cubense* (*Foc*) TR4. (**A** and **C)** were from ‘BX’ cultivar and (**B** and **D)** were from ‘NK’ cultivar at 27 hpi and 51 hpi, respectively. (**A** and **B)** were transverse sections, and (**C** and **D)** were longitudinal sections.

*Foc* usually invades the entire vascular system of pseudostems from damaged roots, eventually reaching the banana fruits, and ultimately damages the yield. Our previous research has verified that *Foc* infects the root tissue after 27 hours and that there are significant metabolic differences between susceptible and resistant cultivars^27^. The early stages of the interaction between banana and pathogen (27–51 h post infection) were used to obtain the expression profiles of lncRNAs in response to *Foc* in banana. In this study, GFP-expressing *Foc* TR4 showed that hyphae and spores had infected the endodermis of the vascular tissues at 27 h and that a greater amount of fungus was found in the cells of ‘BX’ (Fig. 1A) than of ‘NK’ (Fig. 1B). After 51 h, hyphae were found throughout the intercellular space from the infection point, again with more hyphae found in ‘BX’ (average 7-8 hyphae) than in ‘NK’ (average 3-4 hyphae) (yellow arrow) (Fig. 1C,D), which was consistent with the previous report^25^.

### Genome-wide identification of lncRNAs in banana

We performed high-throughput strand-specific RNA-seq in the susceptible ‘BX’ and the resistant ‘NK’ banana cultivars at 27 and 51 hours after inoculation or mock inoculation, with three biological replicates of each combination. From 24 libraries, more than 1.3 billion reads were obtained. All reads were aligned against genes of banana (*Musa accuminata*), with about 60% of the reads mapping to the banana reference genome sequences (Table 1). Among the transcripts, 113,001 transcripts were assembled, of which 107,091 contained one or more exons, and 105,856 transcripts were longer than 200 bp. A total of 95,442 assembled transcripts were completely annotated. After transcripts with very low expression levels were filtered out, 6,345 of the unannotated transcripts were deemed potential lncRNAs. Further filtering was performed using Coding Potential Calculator (CPC), which assessed the quality and completeness of potential ORFs and determined their sequence similarity to proteins in the NCBI protein database. Finally, the remaining transcripts were filtered through the PFAM database. After applying these criteria, 5,294 transcripts were identified as putative banana lncRNAs involved in response to *Foc* (Supplementary Table S1). Of the putative lncRNAs, 85.8% were lincRNA and 14.2% were natural antisense lncRNAs. About 49.7% of all putative lncRNAs were located on the antisense strands.

**Table 1:** Sequencing Metrics of the 24 RNA-seq Libraries.

Source	Library	Total Paired reads	Mapped to genome^1^ (%)
BX-inoculated	27h1	115395024	66715774 (57.82%)
	27h2	105082618	57250387 (54.48%)
	27h3	117369628	72341823 (61.64%)
	51h1	114691296	70672111 (61.62%)
	51h2	100471284	60368380 (60.09%)
	51h3	114125392	68667955 (60.17%)
BX-mock inoculated	27h1	114180298	70896658 (62.09%)
	27h2	103145302	65754668 (63.75%)
	27h3	116999816	70119544 (59.93%)
	51h1	106792164	61916764 (57.98%)
	51h2	113653906	67191970 (59.12%)
	51h3	112149142	69692133 (62.14%)
NK-inoculated	27h1	138481276	84126351 (60.75%)
	27h2	123416978	68739521 (55.7%)
	27h3	126225298	77218929 (61.18%)
	51h1	113247820	70800498 (62.52%)
	51h2	101095984	65529847 (64.82%)
	51h3	117431024	76564366 (65.2%)
NK-mock inoculated	27h1	115028580	75150383 (65.33%)
	27h2	121953596	74191028 (60.84%)
	27h3	116505584	73716059 (63.27%)
	51h1	100662528	61616707 (61.21%)
	51h2	110687792	67928624 (61.37%)
	51h3	115598376	71924504 (62.22%)

^1^The genome was from http://banana-genome.cirad.fr/ ^26^.

### Characteristics of banana lncRNAs

**Figure 2:**
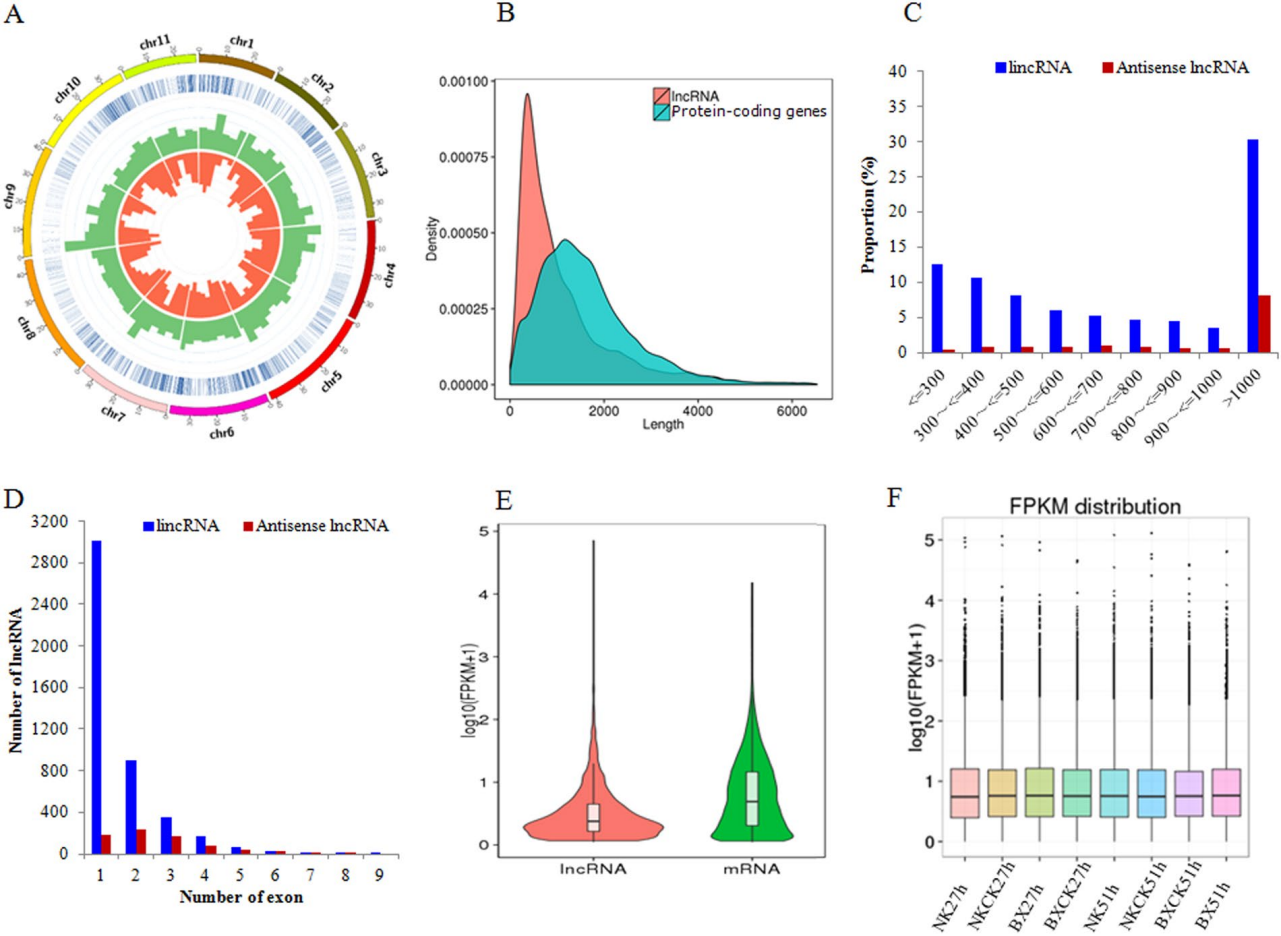
Characteristics of banana lncRNAs. (**A**) Distribution of lncRNAs along each chromosome. The abundance of lncRNA (green) and mRNA (red) are mapped to corresponding coding region on the physical chromosome through Circos. Relative height represents gene expression level. (**B**) Length of all lncRNA and mRNA transcripts on chromosome. (**C**) Length distrubution of 4,544 lincRNAs and 750 antisense lncRNA. (**D**) Distribution of exon numbers of lincRNAs and antisense lncRNAs. (**E**) FPKM distribution of lncRNA and mRNA. (**F**) FPKM distribution of banana lncRNAs under *F. oxysporum* infection.

Banana lncRNAs were preferentially distributed on chromosomes 1, 2, 5, 6, 7, 10 and 11 in the two cultivars (Fig. 2A, Blue circle). The result indicated that the expression trends of most lncRNAs (green bars) were in accordance with those of mRNAs at the corresponding positions of the chromosome (Red bars). The mean lncRNA transcript length was shorter than that for protein-coding genes (1164.87 bp for lncRNA and 1651.2 bp for protein-coding transcripts; Fig. 2B). The lengths of lncRNAs ranged from 201–13848 bp, but more than 61% of the lncRNAs were between 200 and 1000 bp in length, among which lincRNA was more than 90% (Fig. 2C). Approximately 60% of the banana lncRNAs had one exon and 40% had multiple-exons, among which lincRNA with one exon was 56.9% (Fig. 2D) (Supplementary Table S1). Inspection of the global expression normalized to FPKM for all mRNA and lncRNA molecules indicated that the expression levels of most lncRNAs were lower than 10 FPKM (Fig. 2E). Density box plots of banana lncRNA expression (log_10_^(FPKM+1)^) revealed a normal overall distribution of the data points with little systematic bias among the *Foc*- and mock-inoculated expression profiles from different banana cultivars (Fig. 2F) (Supplementary Table S2).

### Validation of transcription levels of banana lncRNAs

To confirm the expression of banana lncRNAs, quantitative Real-Time PCR (qRT-PCR) analysis was applied to verify the results of the high-throughput RNA-seq sequencing. Total RNA extracted from the same samples as RNA-seq used for banana was converted to cDNA by reverse transcription. Totally 22 putative lncRNAs, including 16 lincRNAs and 6 antisense lncRNAs were randomly selected for qRT-PCR validation. Most of the qRT-PCR results reflected the RNA-seq data, with the fold changes from the qRT-PCR and RNA-seq data closely correlated (R^2^ = 0.75, *p* < 0.05) (Supplementary Table S3).

**Figure 3:**
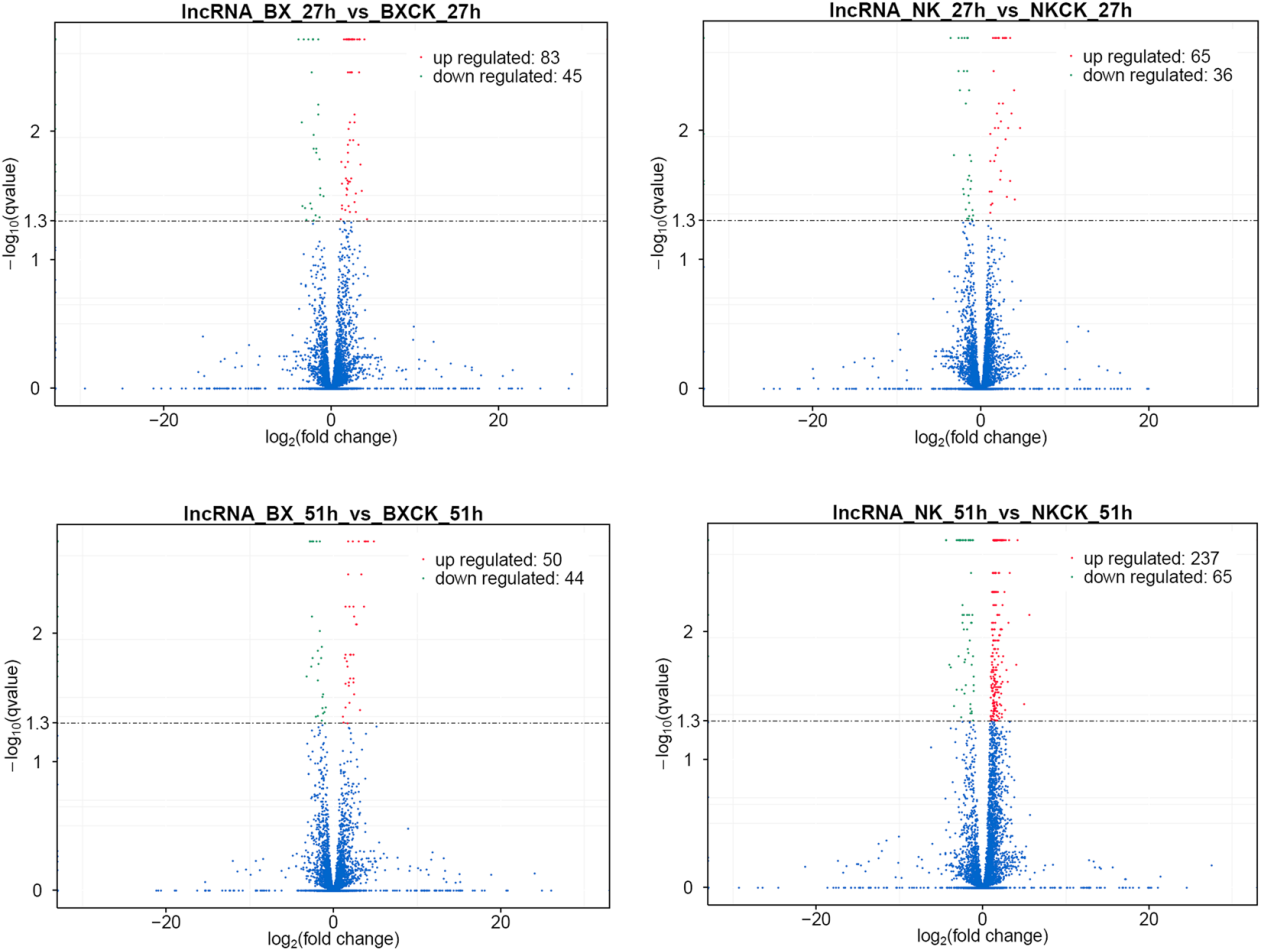
The differentially expressed lncRNAs in two cultivars. The differentially expressed genes (DEGs) were screened with the threshold of *p*-adjust < 0.05 and |Log_2_^(inoculated /mock-inoculated)^| ≥ 1.

LncRNAs with a greater than 2-fold expression change (*p*-value < 0.01 and q-value < 0.05) between the inoculated and mock-inoculated banana were identified as differentially expressed (DE) lncRNAs. The result showed that more DE lncRNAs were more highly induced in ‘BX’ at 27 hpi than at 51 hpi, but it was opposite for ‘NK’ (Fig. 3). There were more DE lncRNAs in ‘BX’ than in ‘NK’ at 27 hpi, but more DE lncRNAs in ‘NK’ than in ‘BX’ at 51 hpi. In addition, 5 and 12 lncRNAs were down-regulated and up-regulated, respectively, in both cultivars at 27 hpi. At 51 hpi, 6 and 12 lncRNAs were down-regulated and up-regulated, respectively, in both cultivars. Of these DE lncRNA, only 3 lncRNAs were up-regulated in both cultivars at two times, including LNC_000010, LNC_002595 and LNC_002624. The different members and the expression profiles of the DE lncRNA in two cultivars implied that they might be response to *Foc* infection by different regulation pathways.

### Functional annotation of the differentially expressed lncRNAs

The regulated genes usually show a consistent or an opposite trend with the regulator gene, and genes and their nearby genes on chromosomes have also been considered to be important for their cis-regulations^18^. So we investigated the potential functions of the DE lncRNAs through mRNAs whose expressions are highly correlated with those of lncRNAs or their nearby mRNAs existing within 100 kb from lncRNAs.

Some pathways were enriched on these mRNAs whose expressions are highly correlated with those of lncRNAs through KEGG analysis (Table 2). The result showed that genes involved in biosynthesis of secondary metabolites, plant-pathogen interaction, phenylpropanoid biosynthesis, and phenylalanine metabolism were greatly induced in the resistant cultivar ‘NK’ at 27 hpi, while genes induced in ‘BX’ were involved in fatty acid metabolism, glycerolipid and glycerophospholipid metabolism, suggesting that ‘NK’ were more effectively response to *F. oxysporum* infection. At 51 hpi, more genes related to galactose metabolism and phenylpropanoid biosynthesis were induced in ‘BX’, while most genes were decreased in ‘NK’. GO enrichment analysis of these genes were shown in Supplementary Table S4. More genes were involved in organic substance metabolic, primary metabolic, biosynthetic, and single-organism metabolic processes in ‘NK’ at 27 hpi than in ‘BX’, while more genes involved in catalytic ativity, transferase activity, and hydrolase activity were found in ‘BX’ than in ‘NK’ at 27 hpi.

**Table 2:** The enriched pathways of mRNAs whose expressions are highly correlated with those of the DE lncRNAs in banana under *F. oxysporum* infection.

Term	ID	Gene numbers			
		NK27h	BX27 h	NK51h	BX51 h
α-Linolenic acid metabolism	mus00592		6, 6up		
Arginine and proline metabo -lism	mus00330		8, 8up		
Biosynthesis of secondary metabolites	mus01110	55, 50up			
Ether lipid metabolism	mus00565		4, 4up	3, 1up	
Fatty acid biosynthesis	mus00061	5, 4up			
Fatty acid degradation	mus00071	5, 5up	6, 6up		
Fatty acid elongation	mus00062			4, 2up	
Galactose metabolism	mus00052		8, 8up	8, 2up	11, 10up
Glycerolipid metabolism	mus00561		10, 10up		
Glycerophospholipid metabolism	mus00564		10, 10up		
Glycosphingolipid biosynthesis - globo series	mus00603		3, 3up	3, 3down	3, 3up
Phenylalanine metabolism	mus00360	14, 12 up			
Phenylpropanoid biosynthesis	mus00940	17, 14up			17, 14up
Plant-pathogen interaction	mus04626	14, 14up			
Sphingolipid metabolism	mus00600		6, 6up	4, 1up	
Ubiquitin mediated proteolysis	mus04120			9, 4up	
Valine, leucine and isoleucine degradation	mus00280	5, 5up	7, 7up	4, 4down	6, 6up

Note: *p*-value < 0.05. The before and after of the comma was the total genes and the up or down regulated genes, respectively, in the pathway.

We also conducted the potential cis-regulation of DE lncRNAs through their nearby mRNA genes (distance < 100 kb) through KEGG analysis (Table 3). More genes were induced in ‘BX’ at 27 hpi than ‘NK’, while at 51 hpi there was fewer pathways were enriched except that genes related to oxidative phosphorylation in ‘NK’. GO enrichment analysis on these nearby genes was shown in Supplementary Table S5 and the distribution of genes were very different between two cultivars and two time points, implying that the pathway enrichment profiles were related to the resposne of banana to *F. oxysporum*.

**Table 3:** The enriched pathways of mRNAs nearby the DE lncRNAs in banana under *F. oxysporum* infection.

Term	ID	Gene numbers		
		NK27h	BX27 h	NK51h
Aminoacyl-tRNA biosynthesis	mus00970	10, 3up		
Brassinosteroid biosynthesis	mus00905		4, 4down	
Folate biosynthesis	mus00790		5, 3up	
mRNA surveillance pathway	mus03015		17, 13up	
Oxidative phosphorylation	mus00190		20, 15up	30, 20up
Peroxisome	mus04146	11, 10up	11, 10up	
Phenylalanine, tyrosine and tryptophan biosynthesis	mus00400		8, 7up	
Proteasome	mus03050		9, 5up	
Protein processing in endoplasmic reticulum	mus04141	26, 14up		
Ribosome	mus03010		41, 30up	
Taurine and hypotaurine metabolism	mus00430		5, 4up	
Vitamin B6 metabolism	mus00750	4, 4up		

Note: *p*-value < 0.05. The before and after of comma was the total mRNA genes and the up or down regulated genes, respectively, in the pathway.

### Expression profiles of DE lncRNAs in plant-pathogen interactions during *Foc* infection

**Figure 4:**
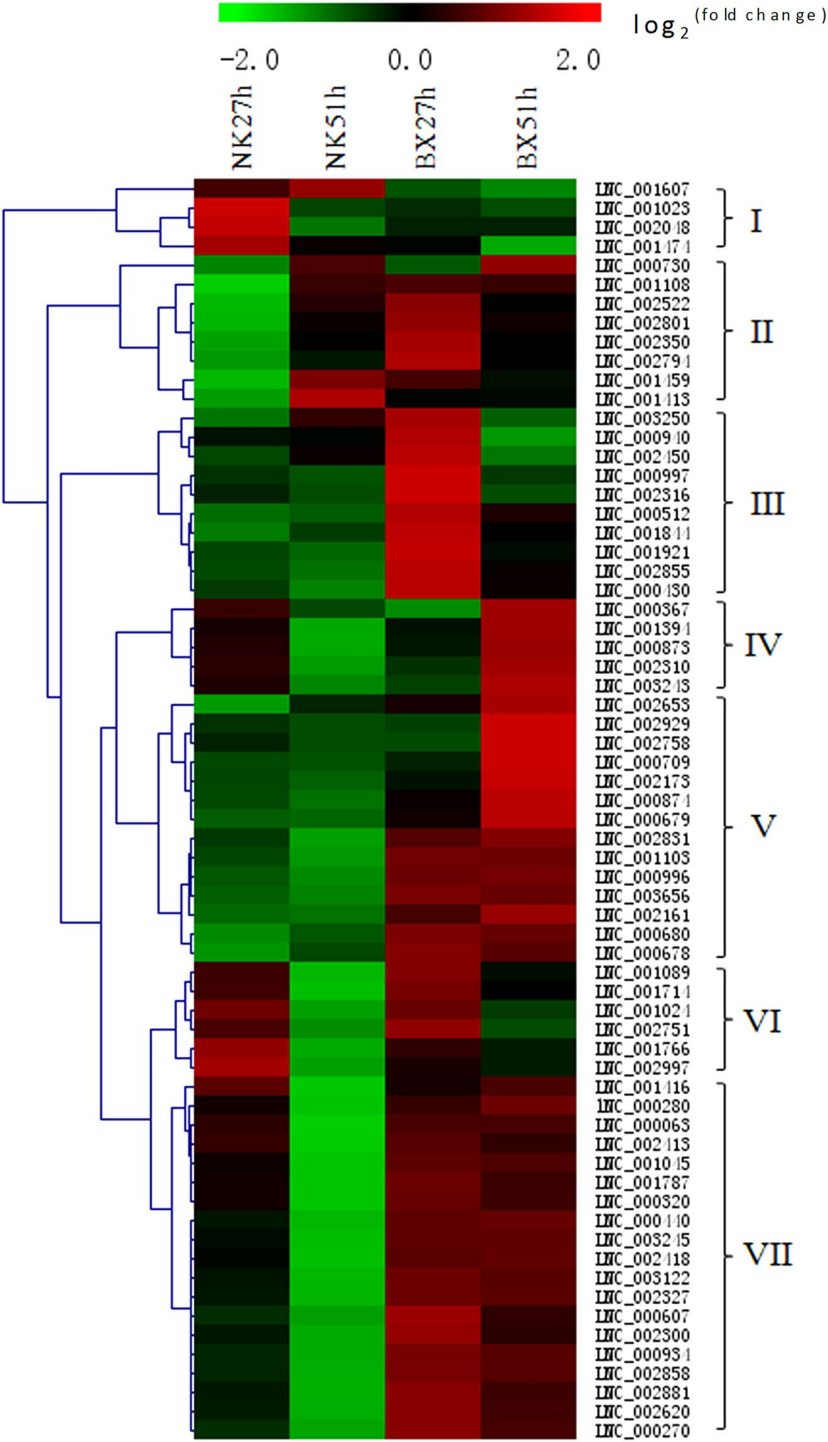
Expression graph of lncRNAs with the potential functions related to plant-pathogen interaction in banana during *F. oxysporum* infection. These lncRNAs have the high expression correlationship with mRNAs related to plant-pathogen interaction. Each rows represents one candidate lncRNA. The expression fold change of the infected over mock-inoculated plant is represented by a color scale ranging from saturated green (−2) to saturated red (2).

More DE lncRNAs that have high expression correlationship with mRNA genes involved in the fungal PAMP-triggered immunity pathway, and encoding pathogenesis-related (PR) protein, thaumatin-like protein, peroxidase, chitinase, defensin and endo β-1,3-glucanase, were analyzed in this study (Supplementary Table S6). These lncRNAs were sorted into seven groups according to their expression profiles (Fig. 4). It was obvious that most of these lncRNAs were induced in the infected ‘BX’. Specifically, lncRNAs clustered in group I were induced in infected ‘NK’ bananas at 27 hpi, and most of them had high expression correlationship with genes encoding peroxidase and pathogenesis-related (PR) proteins. LncRNAs in group II were mainly induced in the infected ‘BX’ samples at 27 hpi, and they also were induced in the infected ‘BX’ and ‘NK’ at 51 hpi. Most of these lncRNAs had high expression correlationship with genes coding all proteins mentioned above for the interaction of plant-pathogen. In group III, lncRNAs were specifically expressed in the infected ‘BX’ at 27 hpi, implying that these lncRNA in ‘BX’ responsed to the pathogen very quickly. In group IV and V, lncRNAs were greatly induced at the later stage of infection in ‘BX’, i.e. 51 hpi. These lncRNA had high expression correlationship with genes encoding all resistant proteins. LncRNAs in group VI were induced in both infected cultivars at the early infection stage of 27 hpi, and most of them had high expression correlationship with genes related to the PR1-like genes. LncRNAs in group VII were mainly induced in the infected ‘BX’, and they had high expression correlationship with genes encoding peroxidase and PR proteins.

The potential cis-regulation of lncRNA through their nearby mRNA genes (within 10 kb) involved in plant-pathogen interactions were considered more important in our study (Supplementary Table S7). About half of all lncRNAs were greatly induced in the infected ‘BX’, including lncRNAs in groups II, III, and IV, and especially more lncRNAs were mainly induced at 51 hpi in ‘BX’ than in ‘NK’, which was similar with those in Fig. 4 (Supplementary Fig. S1). In group I, lncRNAs were induced in the infected samples except for the infected ‘BX’ at 51 hpi, and their nearby genes were related to thaumatin-like protein, chitinase, calmodulin-like protein and defensin. In group V, most lncRNAs were induced in the infected ‘NK’ and some of them were also induced in the infected ‘BX’ at 51 hpi, and their nearby genes encoded calcium-dependent, thaumatin-like, and chitinase-like proteins. Most of lncRNAs in group VI were mainly induced in the infected ‘NK’ at 51 hpi and some of them were also increased in the infected ‘BX’ at 51 hpi.

We validated the expression levels of some lncRNAs and coding mRNAs through qRT-PCR. It was obvious that the expression levels of lncRNA and their high expression correlationship mRNAs were closely correlated (R^2^ = 0.76) (Supplementary Fig. S2), while the correlation of lncRNAs with their nearby mRNAs was low (Supplementary Fig. S3).

### Expression profiles of lncRNAs in phytohormone signal transduction and biosynthesis in banana under *Foc* infection

**Figure 5:**
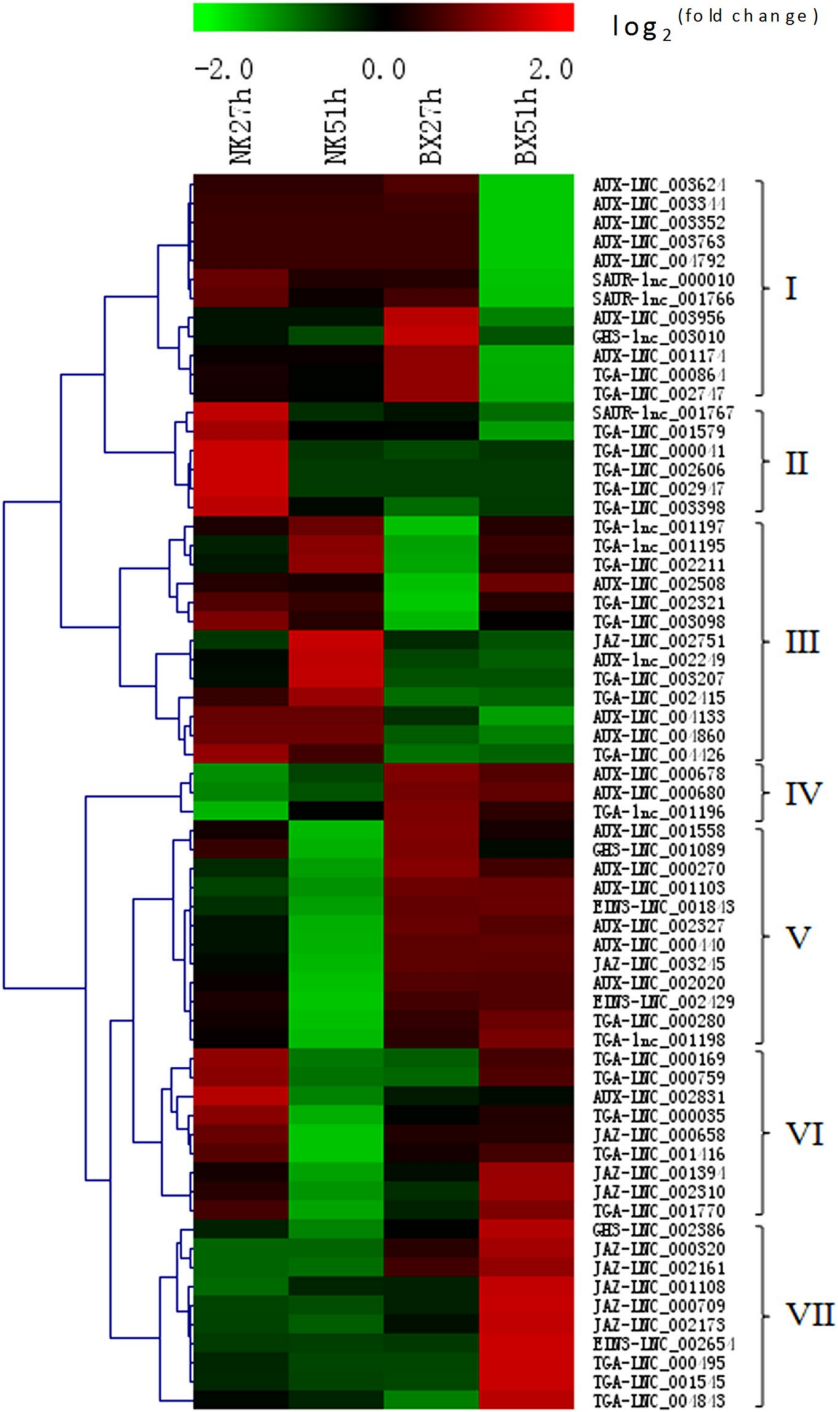
Expression graph of lncRNAs with the potential functions related to phytohormone signal transduction in banana during *F. oxysporum* infection. Each rows represents one candidate lncRNA. The expression fold change of the infected over mock-inoculated plant is represented by a color scale ranging from saturated green (−2) to saturated red (2). ‘LNC’ for lncRNAs with high expression correlationship mRNA related to phytohormone signal transduction. ‘lnc’ for lncRNAs with nearby mRNAs within the distance of 10 kb. AUX: AUX/IAA; GH3: auxin responsive GH3 gene family; SAUR: SAUR family gene; EIN3: ethylene-insensitive protein 3.

Phytohormones are intimately related to the response of plants under biotic and abiotic stresses. We found many DE lncRNAs might be involved in salicylic acid (SA), jasmonic acid (JA), ethylene, and auxin signal transduction through their high expression correlated and nearby coding genes (Supplementary Table S8). The expression hierarchical results showed that lncRNAs in group I were greatly induced in all infected samples except for the infected ‘BX’ at 51 hpi, suggesting that auxin and SA might be very active in the early infection stage due to their high expression correlationship mRNAs were linked to auxin-responsive protein IAA (AUX/IAAs) and transcription factor TGAs (TGAs) (Fig. 5). LncRNAs in group II and III were mainly induced in the infected ‘NK’ at 27 hpi and 51 hpi, respectively, and these lncRNAs might be related to the response of ‘NK’ to *Foc*. However, lncRNAs in group IV and V were greatly induced in the infected ‘BX’ than in the infected ‘NK’, and their high expression correlationship mRNAs were mainly be related with all four hormones signal transduction pathways. For example, the nearby mRNAs of lncRNAs (TGA_lnc_001196 and 001198) encode TGAs in SA signal transduction (Fig. 5). LncRNAs in group VI were greatly induced in the infected ‘NK’ at 27 hpi and the infected ‘BX’ at 51 hpi and most of their high expression correaltionship mRNAs were related to the signal transduction of SA and JA. There were 10 lncRNAs only greatly induced at 51 hpi in the infected ‘BX’ in group VII and their high expression correlationship mRNAs mainly encoded TGAs in SA transduction and jasmonate ZIM domain-containing proteins (JAZ) in JA transduction.

### Concentrations of SA and JA in banana under *Foc* infection

The concentrations of SA and JA were investigated further from 3 to 51 hpi in ‘BX’ and ‘NK’. We otained some DE lncRNAs that had high expression correlationship with their nearby mRNA genes encoding key biosynthetic enzymes of SA and JA.

**Figure 6:**
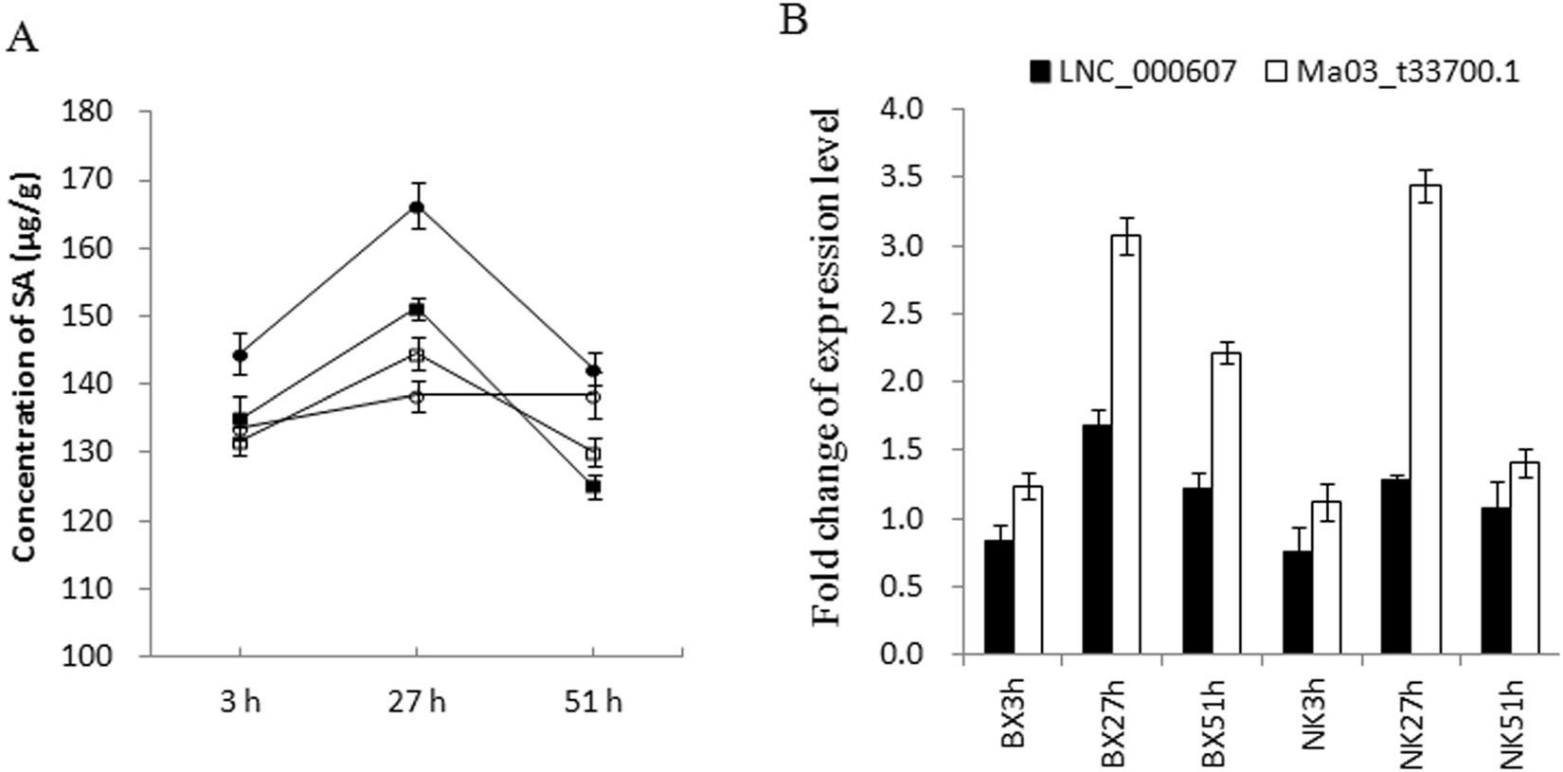
Concentration of SA and the expression levels of potential lncRNAs and coding RNAs related to SA biosynthesis during banana by *F. oxysporum* infection. (**A**) Concentration of SA was measured by HPLC-MS. Error bars represent SE from 6 samples. fwt, fresh weight. Infected ‘BX’ (filled square) and mock-inoculated ‘BX’ (open square). Infected ‘NK’ (filled circle) and mock-inoculated ‘NK’ (open circle). (**B**) qRT-PCR validation of lncRNA and its co-located and potential target gene, isochorismate synthase, ICS. The Y axes are the relative expression abundance from three biological replicates. Bars indicate ± standard error.

The highest concentrations of SA occurred at 27 hpi in both cultivars. The increase in SA content in the infected ‘NK’ was as high as 1.2-fold over the mock-inoculated ‘NK’ at 27 hpi (Fig. 6A, circles), and was nearly 1.1-fold over the infected ‘BX’. At 51 hpi, the content of SA decreased greatly in all mock- or inoculated cultivars, but the content of SA in the infected ‘NK’ was still 1.13-fold higher that in the infected ‘BX’. Interestingly, SA was lower in the infected ‘BX’ compared to the mock-inoculated ‘BX’ at 51 hpi (Fig. 6A, squares). LNC_000607 existed 2605 bp upstream of Ma03_t33700.1, which encodes isochorismate synthase (ICS), and the expression levels of both transcripts were consistent with the changes in SA concentration in both cultivars from 3 to 51 hpi (Fig. 6B).

**Figure 7:**
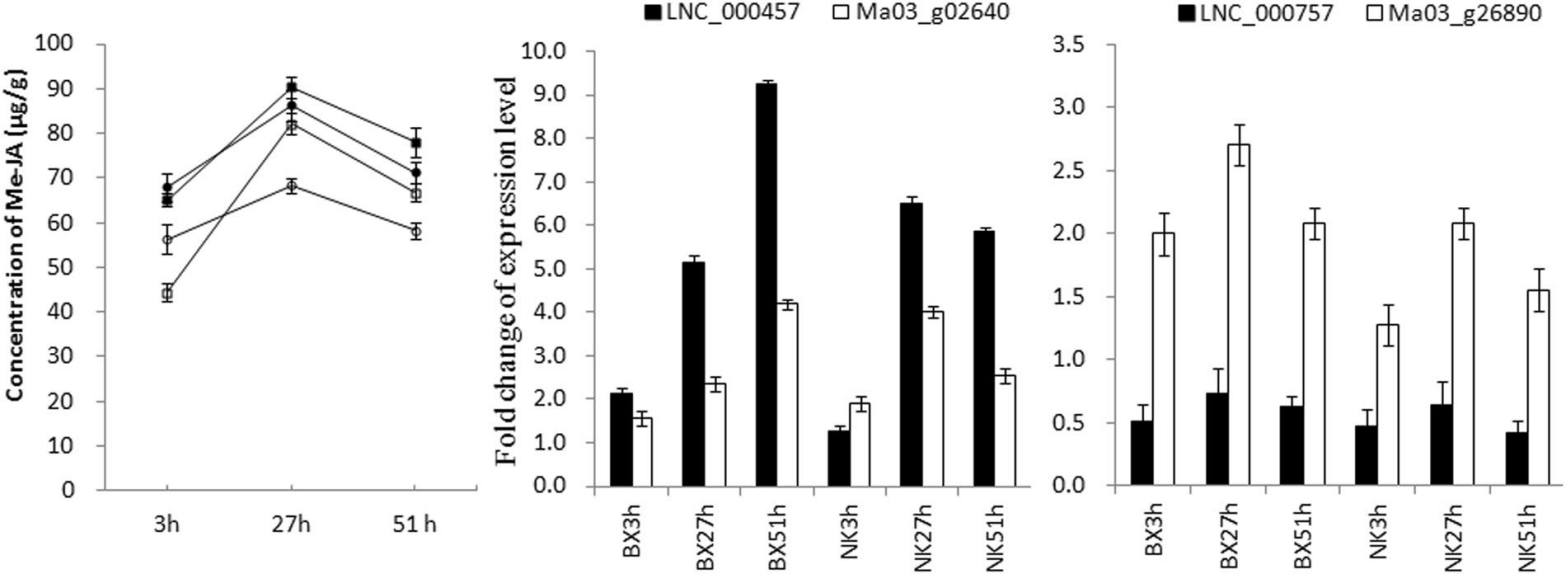
Concentration of JA and the expression levels of potential lncRNAs and coding RNAs related to JA biosynthesis during banana by *F. oxysporum* infection. (**A**) Concentration of SA is from HPLC-MS. Error bars represent SE from 6 samples. fwt, fresh weight. Infected ‘BX’ (filled square) and mock-inoculated ‘BX’ (open square). Infected ‘NK’ (filled circle) and mock-inoculated ‘NK’ (open circle). (**B**) Validation of lncRNA and its co-located, potential target gene through qRT-PCR. AOS, allene oxide synthase. OPR, 12-oxophytodienoate reductase. The Y axes are the relative expression abundance from three biological replicates. Bars indicate ± standard error.

The content of Me-JA was higher in the infected ‘BX’ than in the infected ‘NK’ at all time points (Fig. 7). At 27 hpi, Me-JA showed a peak concentration in all banana plants, however, the level of Me-JA in the infected ‘NK’ was 1.3-fold higher than the level in mock-inoculated ‘NK’. This was a great than seen in the infected and mock-inoculated ‘BX’ (1.1-fold). At 51 hpi, the concentration of ME-JA in all samples decreased, however, the Me-JA was still higher in ‘NK’ compared to ‘BX’. LNC_000457 is 1629 bp downstream of a gene that encodes a 12-oxophytodienoate reductase (Ma03_g02640). Ma03_g02640 showed expression changes consistent with the changes in JA content, and the expression levels of LNC_000457 were higher those of Ma03_g02640 from 3 h to 51 h after infection. The expression of Ma03_g26890, which encodes allene oxide synthase, and LNC_00757 (2455 bp downstream of Ma03_g26890) showed a strong relationship with the content of JA, and the expression levels of Ma03_g26890 were higher those of LNC_00757 during the whole infection in banana.

## Discussion

*Fusarium* wilt disease causes disastrous losses to banana in many tropical and subtropical countries in Asia and Australia^24^. Control measures targeting *Foc*, such as fungicides, crop rotation, fumigation, or antagonistic microbes, can limit disease damage, but epidemics still occur, especially when banana crops are continued within an infected plantation. There is no effective method to control this pathogen. There were no *Foc* TR4-resistant cultivars until somaclonal variants were identified, such as ‘NK’^25^ and ‘Tai Chiao No. 1’^24^. These cultivars present an attractive opportunity to research the resistant and susceptible mechanisms of banana to *Foc* TR 4.

In this study, we used a strand-specific RNA-seq approach to identify and analyze the response of lncRNAs in banana to *Foc* TR4 attack. This approach allowed us to uncover a relatively robust list of potential lncRNAs for banana. Among these 5,294 putative lncRNAs, 162 (3.06%) matched previously reported banana lncRNAs from analysis on banana genome database (http://greenc.sciencedesigners.com/) (Supplementary Table S9)^28^. This suggested that our high throughput sequencing may also not include all lncRNAs in banana, and rare or transient lncRNAs and some lncRNAs responsive to special development stage were not identified under our experimental conditions, since lncRNAs are often processed into smaller, noncoding RNAs^29^. Our study discovered that the amount of lncRNAs should be related to the temporal and spatially specificity.

LncRNAs play important roles in various biotic and abiotic stress in plant. The importance of lncRNA has been emphasized in many species, however, still remained completely unknown until this study in banana under *Foc* infection. Despite obtaining 27 billion RNA-seq reads, it is worth noting that we not only indicated the number of lncRNAs and reported their expression profiles under the inoculation of *Foc* TR4 in banana, but also their potential functions through the high expression correlated and nearby coding mRNAs. We also found that a higher percentage of lncRNAs exhibited *Foc*-specific expression, particularly the DE lincRNAs, in the inoculated versus the mock-inoculated banana and in the susceptible versus the resistant banana. This set of lncRNAs and their expression levels will be useful for functional genomics research or for analysis of potential functional differences among banana varieties. For instance, LNC_000010, LNC_002595 and LNC_002624 were up-regulated in both two cultivars and at two times. Their potential function were involved in catalytic activity, oxidoreductase activity, ethylene-response, cytochrome P450 and so on. The expression levels and the members of the DE lncRNAs determined the response of plant to pathogen infection.

Even though some lncRNAs have verified functions, the molecular mechanism of how lncRNAs participate in regulation process is still largely unknown. LncRNAs can regulate coding genes at transcription, post-transcription, and post-translation levels^4^. They can also modulate the nearby genes positively or negatively by inducing chromatin remodeling or inhibiting RNA polymerase II recruitment^30,31^. So many potential lncRNAs related to plant-pathogen interaction or phytohormone signal transduction were predicted through analysis on the expression profiles, including the expression correlationship coding mRNAs, and nearby coding mRNAs. For instance, LNC_001023, LNC_002048 and LNC_001474 might be related with pathogenesis-related protein and peroxidase due to their high expression correlationship mRNAs and they were specifically more induced in the resistant cultivar ‘NK’ at 27 hpi than in the susceptible cultivar ‘BX’ in group I (Fig. 4), suggesting that they might be related to the response of different cultivars to *Foc*. It was obvious that more lncRNAs were induced at 27 hpi in the plant-pathogen interaction, suggesting that plant respond to pathogen very quickly once Foc infiltrate into plant. Based on the annotation of mRNAs, lncRNAs related to auxin and SA signal transductions might predominantly be induced in ‘BX’, while lncRNAs related to all phytohormes might be induced in ‘NK’. Auxin homeostasis directly links with stress adaptation through interactions with SA and abscisic acid signals. An auxin-deficient *Arabidopsis* mutant showed resistant to both biotic and abiotic stresses^32^. In this study, the result that more lncRNAs with coding genes involved in the auxin transduction were induced in ‘BX’ at the early infection stage might be related with the susceptible of ‘BX’. Furthermore, some lncRNAs with a high expression correlationship with JAZ, a negative regulator of JA signal transduction, were induced in the infected ‘BX’, suggesting that an upregulation of JAZ might inhibit the transduction of JA and ultimately compromise the resistance of ‘BX’ to *Foc*.

From the analysis on the expression correlatioship and nearby genes, one lncRNA usually has many candidate mRNAs. For instance, many lncRNAs are involved in not only the plant-pathogen interaction, but also the phytohormone signal transductions, including LNC_001416, LNC_001766, LNC_002310, LNC_001089, LNC_001103, LNC_001394, LNC_000280 and LNC_003245. On the other hand, many lncRNAs also had the same nearby gene. For instance, the coding mRNA gene Ma05_g24210 had seven lncRNAs within 5000 bp, including LNC_001397, LNC_001398, LNC_001399, and so on. However, our analysis should benefit the prediction of the potential functions of these lncRNAs and their function will need be verified in the future.

Plant lncRNAs may function as competing endogenous RNAs (ceRNAs), by binding to specific miRNAs via target mimicry to protect the miRNA targets^3,33^. We found thirteen lncRNAs (LNC_004963, LNC_005166, LNC_002286, LNC_002287, LNC_002288, LNC_002478, LNC_002479, LNC_002480, LNC_002481, LNC_002482, LNC_002483, LNC_002484, and LNC_002997) that were predicted to be ‘decoys’ for conserved miRNAs, namely mac-nmir12, mac-nmiR20-5p and mac-nmiR3 (Supplementary Table S10)^34^. These microRNAs were novel and their functions are still unknown. Few microRNAs were found to match with the lncRNAs of this study, possibly because the microRNA data was from banana fruit under normal growth condition, while the lncRNAs data was from *Foc*-infected roots. This further shows that lncRNAs have highly specific temporal and spatial expression profiles, which is consistent with previous studies^18,35^.

In conclusion, we obtained 5,294 lncRNAs in banana and reported expression profiles for lncRNAs that were responsive to *F*. *oxysporum* infection in banana. Many *F*. *oxysporum*-induced lncRNAs were associated (through expression correlationship or distance analysis) with genes that have a potential function in disease resistance. Our study demonstrated that lncRNAs are important nodes in the antifungal networks of banana and has provided a foundation for further investigation of the regulatory function of lncRNAs.

## Materials and Methods

### Plant growth conditions and *Foc* inoculation

The *Foc*-susceptible cultivar ‘BX’ and the resistant cultivar ‘NK’ were grown in plastic pots containing nutritious soil in a chamber at 28 °C with a 16 h photo period and a light intensity of 100 μmol m^−2^ s^−1^ for 90 days. The strain *Foc* TR4 VCG01213/16 was isolated from Hainan island of China by Dr. Junsheng Huang (Environment and Plant Protection Institute, Chinese Academy of Tropical Agricultural Sciences, Haikou, China). The strain transformed with GFP was used for inoculation of 90-day-old plants. The root epidermis was either artificially damaged (about 0.5 cm^2^) or uncovered with sterile tweezers and then covered with a freshly prepared *Foc* TR4 block of about 0.5 cm^2^. The *Foc* preparation were per our previous description^27^. For the microscopic examination, banana roots were observed with Laser Microscope (OLYMPUS, FV10-ASW) using filter blocks to select for spectral emission at 488 nm (matching the GFP) and 595 nm (matching root auto-fluorescence)^36^.

### Plant sampling and sample sequencing

Three inoculated and 3 mock-inoculated banana plants, including 3–5 roots per plant, were collected at 27 h and 51 h post inoculation (hpi) and frozen immediately in liquid nitrogen. RNA was extracted from banana roots using plant RNA kit (OMEGA, USA). RNA (3 μg) was used for sample sequencing. Poly(A) RNA enrichment and strand-specific RNA-seq library were prepared using the NEBNext Ultra^TM^ Directional RNA Library Prep Kit for Illumina (NEB, USA) following manufacturer’s recommendations. Library quality was assessed on the Agilent Bioanalyzer 2100 system (Agilent Technologies, CA, USA). Libraries were sequenced on an Illumina Hiseq. 2500 platform with 125-bp paired-end reads.

### lncRNA identification

High quality clean reads were obtained by removing reads with adapter sequences, contaminants or low quality through perl scripts before the downstream analyses. Each RNA-seq clean read was mapped to the banana (*Musa accuminata*) genome (http://banana-genome.cirad.fr.) through TopHat 2.0.9^37^. The transcripts from each library were assembled by Scripture (β2)^38^ and Cufflinks (v2.1.1)^39^. All transcripts were pooled and merged to generate final transcripts using Cuffmerge. Cuffdiff was used to estimate the abundance of all transcripts from the output files of TopHat 2.0^39^. All transcripts without strand information and transcripts that overlapped with known genes were discarded. The remaining transcripts were used to identify the lncRNAs according to a series of strict processes. The transcripts with a FPKM (fragments per kilobase of transcript per million mapped reads) score higher than 0.5 in multiple exons in at least one sample were retained. The transcripts with a length shorter than 200 bp and an open reading frame (ORF) length longer than 120 aa were discarded. Any potential coding of the remaining transcripts was evaluated using Coding Potential Calculator (CPC)^40^ and pfamscan (http://rfam.sanger.ac.uk/) (PFAM)^41^. Only transcripts determined to be non-coding by both CPC and PFAM were considered lncRNAs. The remaining transcripts were searched against the NCBI non-redundant (NR) protein database, KEGG (Kyoto Encyclopedia classification of protein database), COGs (NCBI phylogenetic classification of proteins encoded in complete genomes), and Swiss-Prot (Swiss-Protein database) by BLASTX (E-value cutoff of 1e-10) to exclude transcripts with significant homology to known proteins.

### Gene expression quantification and differential expression analyses

Cuffdiff (v2.2.1) was used to calculate FPKMs and determine differential expression of each lncRNA in each sample^42^. The differentially expressed lncRNA genes (DEGs) were screened with the threshold of *P*-adjust < 0.05 and |Log_2_Fold change| ≥ 1.

### Function analysis of differentially expressed lncRNAs

The potential functions of lncRNA were conducted on high expression correlationship coding genes and nearby coding genes using Gene Ontology (GO)^43^ and KEGG^44^ enrichment analysis. If the pearson correlation value between the coding gene and lncRNA is ≥ 95%, the mRNA gene is considered as high expression correlationship coding genes of lncRNA. The coding mRNAs within 100 kb upstream and downstream of the lncRNA are considered as nearby coding genes. GO terms with corrected P value less than 0.05 were considered significantly enriched by differential expressed lncRNAs. The GO annotaions were functionally classified by WEGO software for gene function distributions. KOBAS software was used to test the statistical enrichment of differential expression genes in KEGG pahtways. The pathways with an FDR value of ≤ 0.05 were defined as those with genes that display significant levels of differential expression.

### Quantitative real-time PCR validation of RNA-Seq data

Quantitative RT-PCR (qRT-PCR) primers for the lncRNAs and mRNA genes were designed using Primer Premier software (6.0) based on the gene sequence information (http://banana-genome.cirad.fr/) (Supplementary Table S3). Reactions were performed on an Applied Biosystems StepOne Real-Time PCR system with a 96-well plate (Applied Biosystems, Foster City, CA, USA) in a final volume of 20 μl 2 × SYBR Premix ExTaq^TM^ II Kit (TaKaRa, Dalian, China).

The PCR reaction was: 95 °C for 30 s, followed by 40 cycles of 5 s at 95 °C, and 30 s at 58 to 60 °C. At the end of each experiment, a melt-curve analysis was performed using the default parameters (15 s at 95 °C, 1 m at 55 °C to 95 °C in 0.3 °C increments, and 15 s at 95 °C). The relative expression levels of the target genes were calculated by the 2^−ΔΔCt^ method^45^. β-actin gene and glyceraldehydes-3-phosphate dehydrogenase 2 (GAPDH) were employed as internal references to normalize the transcriptional levels of target genes.

### Determination of salicylic acid and jasmonic acid

Salicylic acid (SA) and methyl-jasmonic acid (Me-JA) were measured using modified method^27,46^. Briefly, 6 g of ground fresh banana roots were extracted with 20 ml of 80% (v/v) methanol containing 1% acetic acid (v/v) for 16 h at 4 °C. After centrifugation, the supernatant was extracted using a 3:1 mixture of 0.2 M Na_2_HPO_4_:H_3_PO_4_ (v/v) and 3 ml petroleum ether at 4 °C for three times. The water phase was adjusted to pH = 8.0 by Na_2_HPO_4_ and was twice extracted with an equal volume of ethyl acetate. The ester phase was evaporated at 10 °C and dissolved in 50% methanol (v/v) to 1 ml for LC/MS analysis.

## Electronic supplementary material

Supplementary figures

Supplementary tables
