# Supplementary material for: Long noncoding RNAs that respond to Fusarium oxysporum infection in ‘Cavendish’ banana (Musa acuminata)
Source: Sci Rep. 2017 Dec 5;7:16939. doi: 10.1038/s41598-017-17179-3 (PMC5717134; doi:10.1038/s41598-017-17179-3)

Supplementary materials

Long noncoding RNAs that respond to *Fusarium oxysporum* infection in 'Cavendish' banana (*Musa acuminata*)

Wenbin Li, Chunqiang, Li, Shuxia Li, Ming Peng*

Supplementary Fig. S1. The expression levels of lncRNA with their nearby mRNA genes involved in plant pathogen interaction in banana under *F. oxysporum* infection.

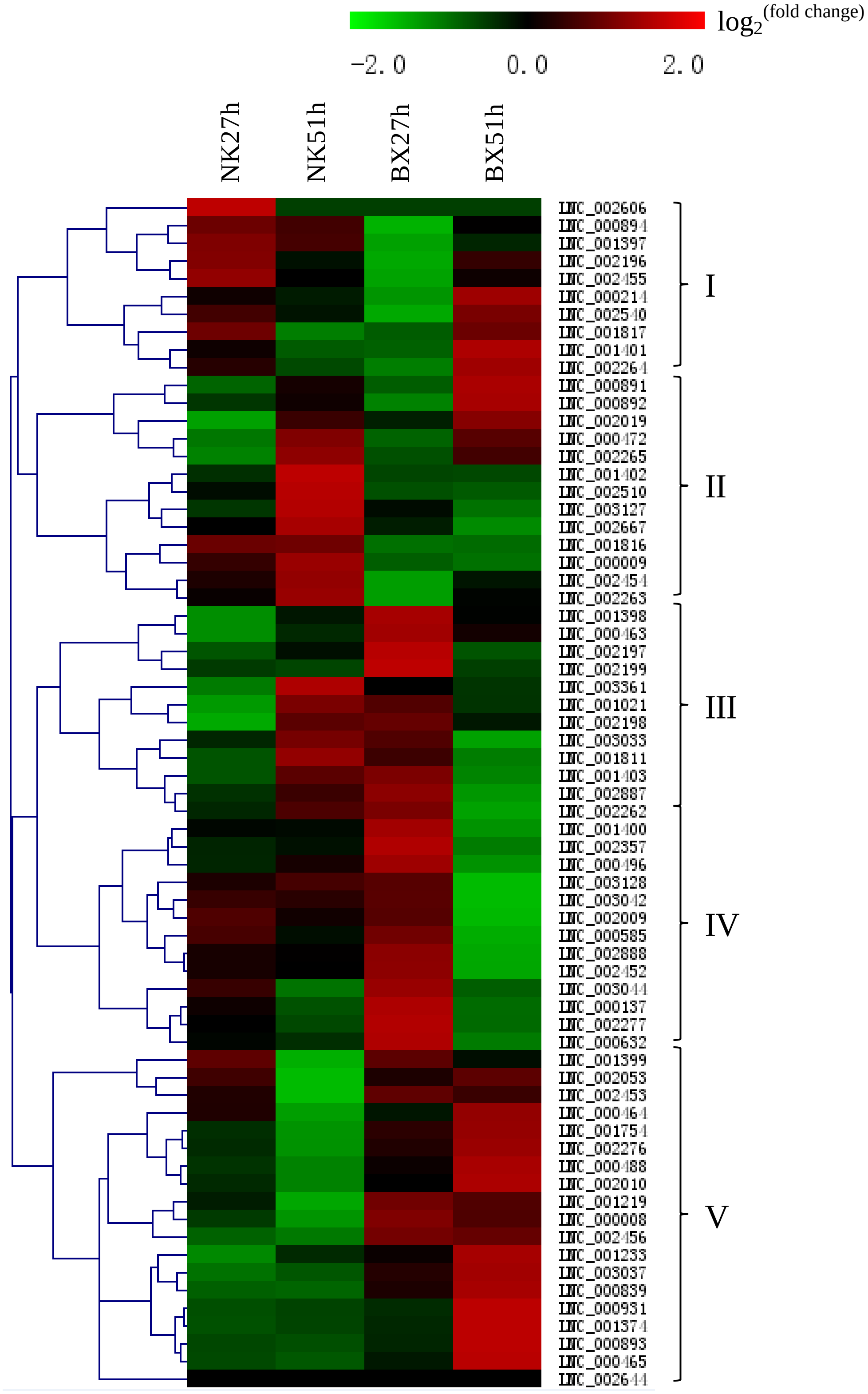

Each rows represents one candidate lncRNA. The expression fold change of the infected over mock-inoculated plant is represented by a color scale ranging from saturated green (-2) to saturated red (2).

Supplementary Fig. S2. The expression levels of lncRNA and their high expression coorelationship mRNAs related to plant pathogen interaction in banana at 27 and 51 hours after *F. oxysporum* infection.

The Y axes are the expression fold change of genes in infected plants over the mock-inoculated plants from three biological replicates.

Bars indicate ± standard error. The X axes are banana cultivar and innoculation time.

R square value: the correlationship between the expression of lncRNA and mRNA genes from the correl function.

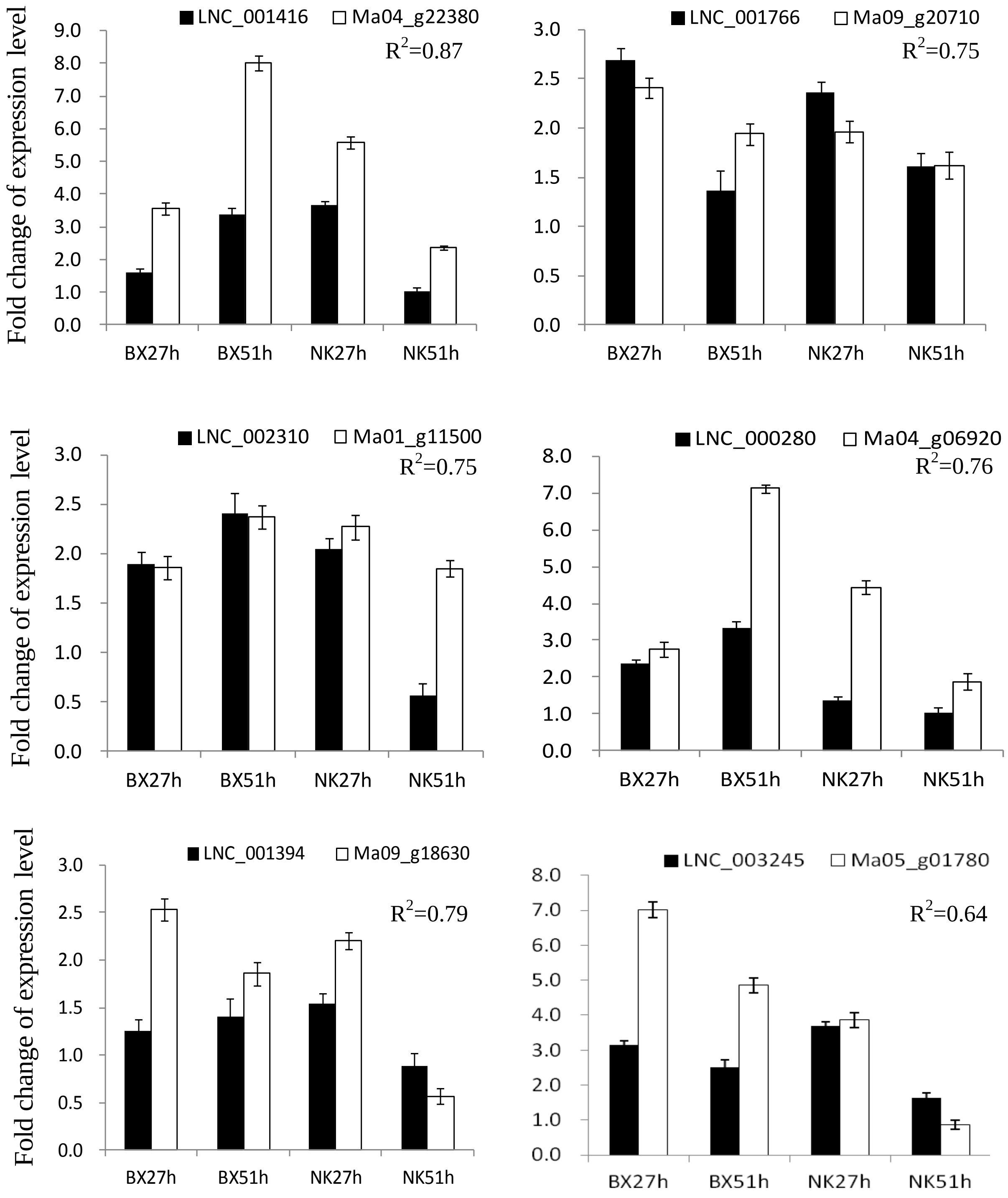

Supplementary Fig. S3. The expression levels of lncRNA and their nearby mRNAs related to plant pathogen interaction in banana at 27 and 51 hours after *F. oxysporum* infection.

The Y axes are the expression fold change of genes in infected plants over the mock-inoculated plants from three biological replicates.

Bars indicate ±standard error. The X axes are banana cultivar and innoculation time.

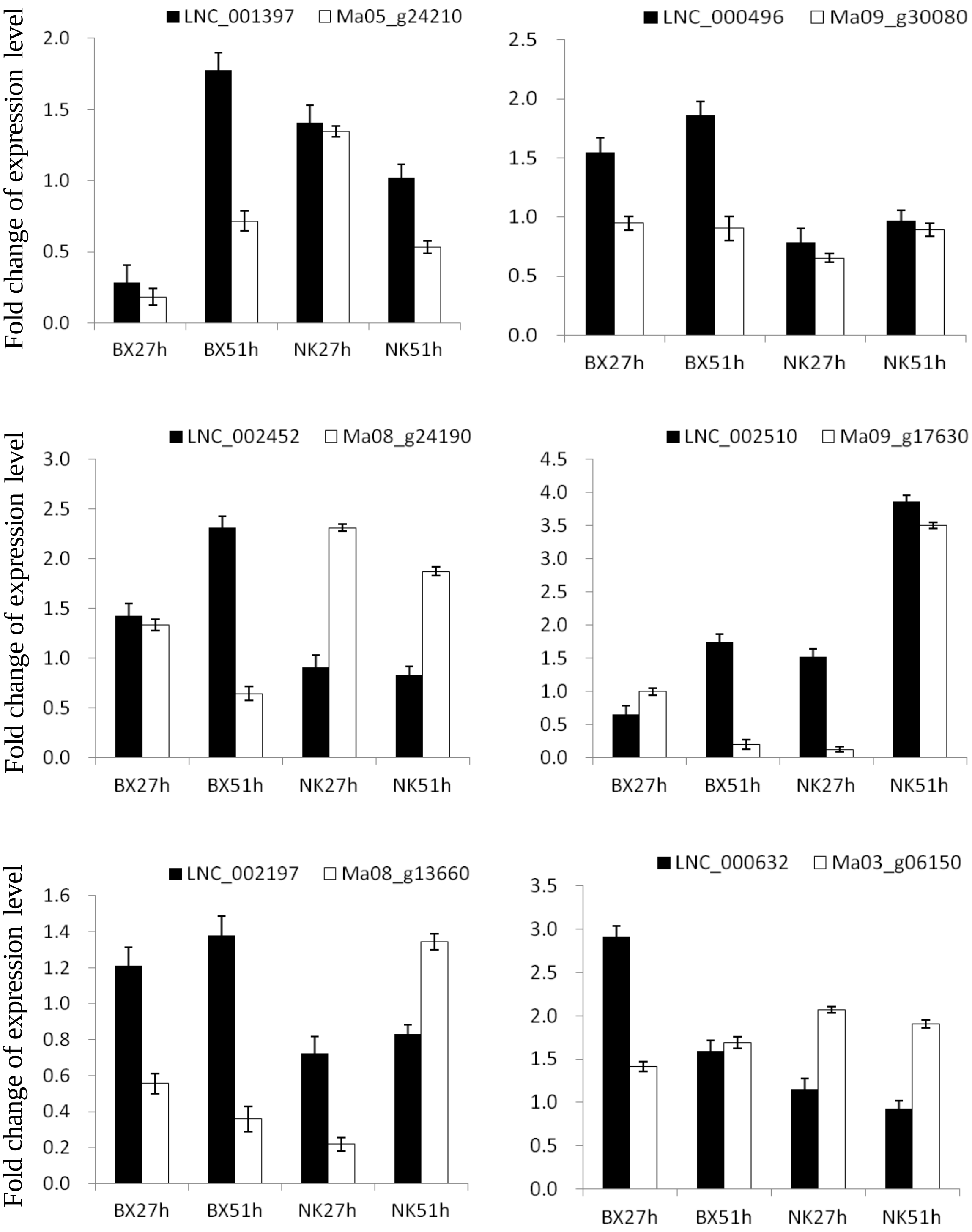

Supplement: Supplementary file 1 — Supplementary figures [file 41598_2017_17179_MOESM1_ESM.pdf]
